# Stable Perovskite Quantum Dots Light‐Emitting Diodes with Efficiency Exceeding 24%

**DOI:** 10.1002/advs.202304696

**Published:** 2023-10-27

**Authors:** Xuanyu Zhang, Qiangqiang Wang, Zhiwei Yao, Ming Deng, Jing Wang, Lei Qian, Yong Ren, Yuying Yan, Chaoyu Xiang

**Affiliations:** ^1^ Laboratory of Advanced Nano‐Optoelectronic Materials and Devices Ningbo Institute of Materials Technology and Engineering Chinese Academy of Science Ningbo Zhejiang 315201 China; ^2^ Nottingham Ningbo China Beacons of Excellence Research and Innovation Institute Ningbo 315040 China; ^3^ Faculty of Engineering University of Nottingham Nottingham NG7 2RD UK; ^4^ Division of Functional Materials and Nanodevices Ningbo Institute of Materials Technology and Engineering Chinese Academy of Sciences Ningbo 315201 China; ^5^ Laboratory of Advanced Nano‐Optoelectronic Materials and Devices Qianwan Institute of CNITECH Ningbo 315300 China; ^6^ Zhejiang Provincial Engineering Research Center of Energy Optoelectronic Materials and Devices Ningbo Institute of Materials Technology & Engineering Chinese Academy of Science Ningbo Zhejiang 315201 China; ^7^ Key Laboratory of Carbonaceous Wastes Processing and Process Intensification Research of Zhejiang Province University of Nottingham Ningbo China Ningbo 315100 China; ^8^ Department of Electrical and Electronic Engineering University of Nottingham Ningbo China Ningbo 315100 China; ^9^ School of Mechanical Engineering and Mechanics Ningbo University Ningbo Zhejiang 315211 China

**Keywords:** LEDs, narrow emission, perovskite nanocrystals, stability, synthesis

## Abstract

Perovskite nanocrystals for light‐emitting diodes are often synthesized by uncontrollable metathesis reactions, suffering from low product yield, nonuniform growth, and poor stability. Herein, by controlling the nucleation kinetics with high dissociation constant (Ka or Kb) acids or bases, homogenous one‐route nucleation of perovskite nanocrystals is achieved as the cluster intermediates are eliminated. The stable, shape uniform, and narrow size distribution green nanocrystals are synthesized. The perovskite nanocrystal film exhibites excellent stability in 80% humidity air with only a 10% photoluminescence intensity drop after 16 h. Efficient and stable electroluminescence is demonstrated with an FWHM of 16 nm at 517 nm. The green devices shows a peak EQE of 24.13% with a lifetime T_50_ of 54 min at 10 000 cd m^−2^.

## Graphical abstract

Stable green perovskite nanocrystals are synthesized by a modified hot injection method, which control the nucleation and growth process with short‐chain length acids/bases and resurfacing agents. The fabricated green perovskite QLEDs achieves operation stability T_50_ of 54 min at 10 000 nits with high efficiency exceeding 24%. The peak emission is 517 nm with FWHM of 16 nm.

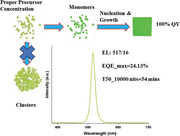

## 1 Introduction

Lead halide perovskite nanocrystals (LHP NCs)are promising emitting materials^[^ ^1^, ^2^ ^]^ that can achieve near‐unit photoluminescence quantum yield (PLQY),^[^ ^3^, ^4^ ^]^ narrow spectra,^[^ ^5^, ^6^ ^]^ and easy wavelength tunability.^[^ ^7^, ^8^ ^]^ With their low‐cost solution methods for thin film fabrication,^[9^ ^]^ the LHP NCs show great potential for photoelectronic applications, such as light‐emitting diodes (LEDs),^[^ ^9^, ^10^, ^11^ ^]^ lasers,^[^ ^12^ ^]^ and radiation scintillators.^[^ ^13^ ^]^ However, perovskite NCs easily degrade or merge into the bulk phase, resulting in severe emission quenching. It is still a challenge to synthesize perovskite NCs that can keep stable in the air and under continuous UV illumination.^[^ ^14^ ^]^

**Figure 1:**
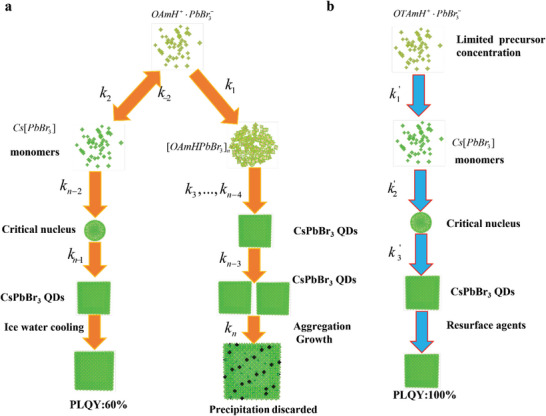
Overall reaction paths of hot injection method for LHP nanocrystal synthesis a)reaction path using OA/OAm as surfactants and b)improved reaction path introducing OTAc/OTAm as extra surfactants. where k_1,_…, k_n_ and k_1_’,…, k_n_’ represent different reaction kinetic constants.

LHP NCs are composited of ionic chemical bonds with soft lattice,^[^ ^14^ ^]^ while conventional II‐VI or III‐V quantum dots possess covalent bonding and rigid crystal structures.^[^ ^15^ ^]^ For typical synthesis methods, LHP NCs developed too quickly during ionic co‐precipitation.^[^ ^2^, ^14^, ^15^ ^]^ The overall reaction path of typically used oleylamine (OAm) and oleic acid (OA) as ligands are described in **Figure** **1**a. For a typical reaction, in the presence of OAm/OA, PbBr_2_ precursors quickly dissolve as follow^[^ ^16^ ^]^:

(1) $$\begin{array}{c} 2{\mathrm{PbBr}}_{2}+{\mathrm{OAmH}}^{+}\cdots {\mathrm{OA}}^{-}\to {\mathrm{OAmH}}^{+}\cdot {\mathrm{PbBr}}_{3}^{-}+{\mathrm{PbBr}}^{+}\cdot {\mathrm{OA}}^{-} \end{array}$$

(2) $$\begin{array}{c} 3{\mathrm{PbBr}}_{2}+2{\mathrm{OAmH}}^{+}\cdots {\mathrm{OA}}^{-}\to 2{\mathrm{OAmH}}^{+}\cdot {\mathrm{PbBr}}_{3}^{-}+2\mathrm{Pb}{\left(\mathrm{OA}\right)}_{2} \end{array}$$

**Figure 2:**
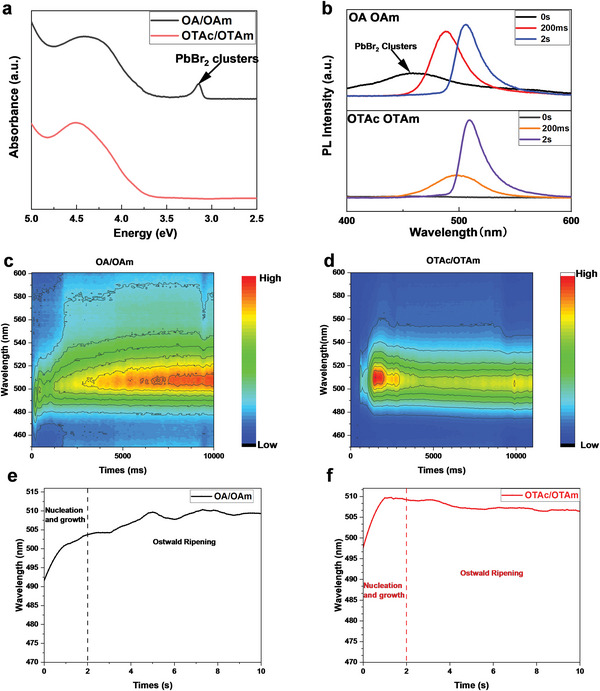
a) absorption spectra of precursor solutions with different surface ligands. b) extracted PL from in‐situ PL measurement, 0 s, 200 ms, 2 s. In situ PL of perovskite NCs nucleation and growth c) OA/OAm, d) OTAc/OTAm. Emission peak evolution of e) OA/OAm and f) OTAc/OTAm based CsPbBr_3_ NCs. Due to the quantum confinement effect, the size of nanocrystal is correlated to its emission peak.

With an ideal ratio of OAm and OA amounts, the lead bromide salt (PbBr_2_) is partially converted into halo plumbate ionic solute (${\mathrm{PbBr}}_{3}^{-}$)^[^ ^9^, ^17^ ^]^ as described by equation (1). Meanwhile, high concentration ${\mathrm{PbBr}}_{3}^{-}$ can also be created where the amount of OAm and OA is much more than the minimum amount required for dissolving PbBr_2_, which is described by equation (2). In this case, the high concentration ${\mathrm{PbBr}}_{3}^{-}$ further produces the cluster intermediates^[^ ^17^, ^18^ ^]^{OAmH^+^ · [PbBr_3_]}_*n*_, which is depicted by the reaction kinetic constant *k_1_* in Figure 1a. The existence of cluster intermediates can be observed as the emerging absorption peak at 400 nm as shown in **Figure** **2**a. After Cs‐oleate is injected, ${\mathrm{PbBr}}_{3}^{-}$ is converted into monomers Cs[PbBr_3_] as *k_2_*, *k_‐2_* described in Figure 1a.^[^ ^15^ ^]^ The monomer immediately reaches the critical concentration and LHP nanocrystal nucleation happens (*k_n‐2_* in Figure 1a). Meanwhile, the cluster intermediates are also converted into CsPbBr_3_ nuclei (*k_3_…k_4_* in Figure 1a). As the result, the nucleation is nonuniform because that the two routes are competing to produce different size nuclei. After Ostwald ripening, large‐size nuclei derived from cluster intermediates grow up (reaction kinetic constant *k_n_*), which suffer from ligand loss, surface defects, low PLQY, and even precipitate due to aggregation.^[^ ^19^, ^20^ ^]^ Thus, the nonuniform nucleation and growth give rise to low yield, broad size distribution, and instability of final NCs.

In previous studies, reaction parameters such as temperature, amount of ligand, molar ratio of precursors and reaction time were investigated for uniformity of perovskite NCs.^[^ ^16^, ^21^ ^]^ However, due to the difficulty of tuning the nucleation and kinetic of perovskite NCs synthesis with OAm and OA, it is hard to improve the NCs uniformity and stability. Meanwhile, due to the limitation of ligands that can be used, the performance of LHP NCs applied to electroluminescence is unsatisfied.^[^ ^11^, ^22^ ^]^ Therefore, the size controllable and highly uniform LHP NCs which can be applied for perovskite quantum dot light‐emitting diodes (PeQLEDs) are still challenging.

Here, to synthesize high‐quality perovskite NCs for PeQLEDs, we propose a strategy as chemical equilibrium‐assisted homogeneous synthesis for uniform nucleation and controllable growth of LHP NCs. Our strategy aims to avoid the creation of PbBr_2_ clusters, thus eliminating the nonuniform nucleation. Together with established thermodynamic equilibrium and resurface agents, the diverse growth after the nucleation is also suppressed. As the dissolution of PbBr_2_ is governed by acid‐base equilibrium,^[^ ^16^ ^]^ high dissociation constant acids and bases form a more stable salt which is less active to dissolve the PbBr_2_.^[^ ^23^ ^]^ The dissociation ability of those stable salts is much lower than oleylamine oleate acid salt. Consequently, they can efficiently control the ${\mathrm{PbBr}}_{3}^{-}$ concentration and prevent the formation of PbBr_2_ clusters. Here, octanoic acid (OTAc) and octylamine (OTAm) are used as the case of high dissociation constant acids and bases to study. The dissolution of PbBr_2_ precursor solely follows the reaction:

(3) $$\begin{array}{ccc} & & 2{\mathrm{PbBr}}_{2}+{\mathrm{OTAmH}}^{+}\cdots {\mathrm{OTAc}}^{-}\\ & & \;\to {\mathrm{OTAmH}}^{+}\cdot {\mathrm{PbBr}}_{3}^{-}+{\mathrm{PbBr}}^{+}\cdot {\mathrm{OTAc}}^{-} \end{array}$$

and avoids the creation of intermediates. The perovskite synthesis then goes through the precursor‐monomer‐QD nuclei conversion path^[^ ^19^, ^24^ ^]^ as described in Figure 1b. Along with the addition of resurfacing agents, the aggregation growth after the nucleation is suppressed by thermodynamic equilibrium. Resurface agents also stabilize quantum dots and passivate surface defects.

With this method, the synthesized LHP NCs showed excellent environmental stability (humidity 80% in air), strong photostability, narrow photoluminescence (PL) emission, and high PLQY. Highly efficient and stable green PeQLEDs were fabricated with narrow emission whose full width of half maximum (FWHM) is only 16.1 nm. The champion device has an external quantum efficiency (EQE) of 24.13%, while the average of 40 devices is 23.10%. The operation stability was dramatically enhanced. For the optimized device, T_50_ lifetime of 54 min at 10 000 cd m^−2^ is achieved.

## 2 Results and Discussion

### 2.1 Synthesis of High‐Quality LHP NCs

The synthesis dynamics of OTAc/OTAm based LHP NCs are compared with the control sample using OA/OAm. Figure 2a is absorption profiles of precursor solutions. For OA/OAm method, there are two absorption peaks at 3.15 and 4.5 eV, which can be attributed to PbBr_2_ clusters and PbBr_2_ precursors respectively. For OTAc/OTAm method, there is only one absorption peak of PbBr_2_ precursors at 4.5 eV, which indicates the prohibition of *k_1_* reaction. The nanocrystal nucleation and growth process were investigated by in situ PL measurement, the results are shown Figure 2c,d. We extracted critical PL spectra from 0 s, 200 ms, 2 s and show them in Figure 2b. For OA/OAm method, the PL peak of PbBr_2_ clusters disappeared after Cs‐oleate was injected, which indicates the clusters were transferred into NCs. For comparison, there was no emission peak in 400 to 460 nm with OTAc/OTAm. The PbBr_2_ clusters were also observed from OA/OAm method as shown in TEM image of supporting information Figure S1a (Supporting Information).

The nucleation and growth can be analyzed through evolution of emission peaks in Figure 2b,e,f. As nucleation and growth cannot be separated clearly,^[^ ^2^, ^15^ ^]^ we can analyze the first 2 s of the process when monomers were not yet exhausted. For OA/OAm synthesis, the NC size increased relatively slowly before the Cs[PbBr_3_] monomers were depleted. The present of PbBr_2_ clusters, which transferred into nucleus after Cs‐oleate injection, results in very low Cs [PbBr_3_] monomer concentration. When Ostwald ripening occurs later, the large size of NCs continues to aggregate^[^ ^25^, ^26^ ^]^ and the size of nanocrystals slowly increased as the emission peak shifted to long wavelength. In comparison, the nuclei of OTAc/OTAm sample grew faster than the OA/OAm sample, which indicated a sufficient monomer supply and a lower nucleus concentration when Cs‐oleate was injected. Then, the nanocrystal size was slightly decreased, suggesting that Ostwald ripening suppressed due to the established thermodynamic equilibrium limitation.^[^ ^27^ ^]^ The XRD result of perovskite nanocrystal film (Figure S2, Supporting Information) proves that OTAc/OTAm LHP NCs have better crystallization. Compared with the OA/OAm based LHP NCs, the OTAc/OTAm LHP NCs show a narrow emission peak indicating more uniform size distribution from the beginning of nucleation until the end of growth (Figure S1b, Supporting Information). Dynamic light scattering (DSL) measurements (Figure S3, Supporting Information) also confirmed the narrower size distribution of OTAc/OTAm LHP NCs.

### 2.2 High Photoluminescent Efficiency and Stability

**Figure 3:**
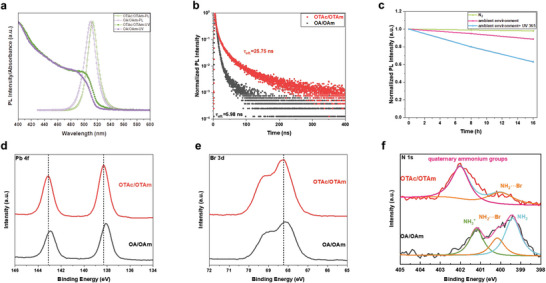
a) PL spectra of synthesized NCs. b)TRPL of synthesized perovskite film. c)PLQY of OTAc/OTAm perovskite NC film in the ambient environment and UV 365. High resolution XPS of OTAc/OTAm and OA/OAm perovskite NC film, d)Pb 4f, e)Br 3d, f) N 1s.

As shown in **Figure** **3**a, the OTAc/OTAm LHP NCs show narrow photoluminescence spectra, which is located at 515 nm with FWHM of 18 nm. The transient photoluminescence (TRPL) performance of the perovskite films is shown in Figure 3b. The effective photoluminescence lifetime of OTAc/OTAm LHP NCs is 25.75 ns, while the OA/OAm nanocrystals is only 5.98 ns. The much longer effective photoluminescence lifetime of OTAc/OTAm LHP NCs indicates that there are fewer defects. The details of TRPL results are listed in support information Table S1 (Supporting Information). The near unity PLQY of the OTAc/OAm NC solution has been measured. The stability of OTAc/OTAm LHP NC films in an ambient environment and under UV light was studied. The OTAc/OTAm perovskite NC film has strong resistance to oxygen, moister and UV light. Figure 3c shows the PL intensity of OTAc/OTAm LHP NC film kept up to 90% after 16 h in the ambient environment (humidity 80%). When the nanocrystal film was continually exposed to UV 365 for 16 h under the same ambient environment, it could keep ≈60% of its initial PL intensity. We also studied the stability of OTAc/OTAm perovskite nanocrystal films under different intensity of UV 365 (Figure S4, Supporting Information). Different In contrast, OA/OAm perovskite nanocrystals films suffer severe fluorescence quenching as shown in Figure S5 (Supporting Information).

The enhanced stability performance can be attributed to the better control of the synthesis as well as the resurface agents. The precursor used for resurfacing agents was synthesized with zwitterionic ligands (ASC18) and HBr. Surface defects, such as Cs and Br vacancies can also be passivated through quaternary ammonium ions and bromide ions in the ASC18‐HBr.^[^ ^3^, ^4^, ^28^, ^29^, ^30^ ^]^ The sulfonate group which has a strong binding with Pb can also passivate the Br vacancies.^[^ ^28^, ^31^ ^]^ According to the FTIR measurements (Figure S7, Supporting Information), OTAc/OTAm LHP nanocrystal film has the 1465 and 1589 cm^−1^ peaks, which can be attributed to C‐H bending and quaternary ammonium groups (antisymmetric deformation of primary amine salt) respectively. The two absorption peaks at 1165 and 1035 cm^−1^ are related to the sulfonic groups (symmetric stretching of SO_2_ and antisymmetric stretching of S‐O). The film XPS measurement demonstrated strong chemical interaction between resurface agents and perovskite NCs. In Figure 3d–f, it is observed that the Pb 4f, Br 3d, and N 1s peaks of OTAc/OTAm LHP nanocrystal film all shift to the higher binding energy compared with the OA/OAm LHP nanocrystal film, indicating that OTAc/OTAm LHP NCs have stronger surface ligands binding. Moreover, for the N 1s peaks of OTAc/OTAm LHP NCs, the intensity of the binding energy peak which is located at 402 eV is stronger than the peak located at 400 eV. The strong peak can be attributed to quaternary ammonium ions. Together with the evidence in FTIR measurements, the OTAc/OTAm nanocrystal surface was better passivated with ASC18. Moreover, the perovskite nanocrystals films exhibited excellent charge mobility compared to OA/OAm nanocrystals films (Figure S9, Supporting Information).

### 2.3 Efficient and Stable PeQLEDs

**Figure 4:**
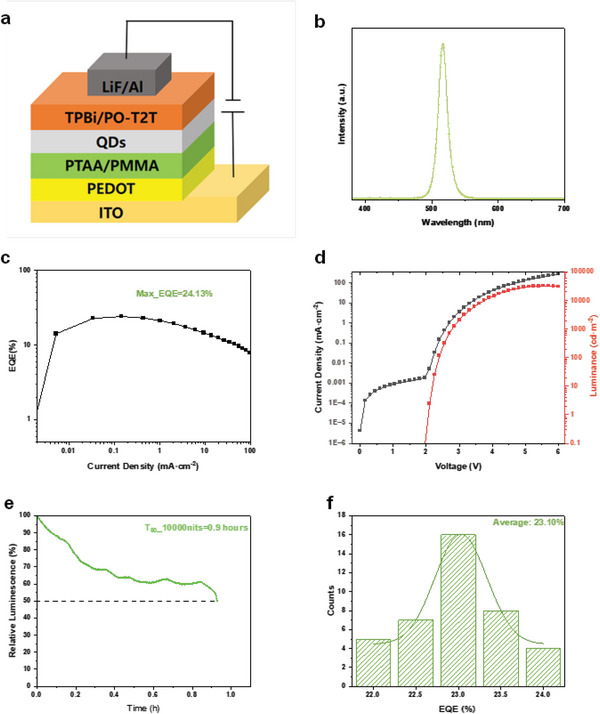
a) Device Structure of perovskite QLED. b) Electroluminescence of Green QLEDs. c) EQE versus current density of green perovskite QLEDs. d) J‐V‐L of perovskite QLEDs. e)Operation stability of perovskite QLEDs. f) EQE distribution of 40 devices.

Efficient and stable PeQLEDs were fabricated using homogenous NCs. The device structure of our green perovskite PeQLEDs was ITO/PEDOT: PSS/PTAA/PMMA/LHP NCs/TBPI/PO‐T2T/LiF/Al, **Figure** **4**a. The maximum external quantum efficiency (EQE) of green PeQLEDs reached 24.13% for the optimized device shown in Figure 4c. We measure angular intensity distribution of fabricated PeLEDs and ensure that our PeLEDs are Lambertian emission (Figure S10, Supporting Information). The average EQE of 40 devices is 23.10% as shown in Figure 4f. The electroluminescence of the optimized device is located at 517 nm with FWHM at 16.1 nm. The operation stability of the champion devices was plotted in Figure 4e. At the initial brightness of 10 000 cd m^−2^, the T_50_ (time when the luminance drops to 50% of initial value) reached 54 min. The acceleration factor measured as 1.7 (Figure S11, Supporting Information). In our knowledge, our devices achieved a new record for PeQLEDs (Table S2, Supporting Information).

## 3 Summary

A homogenous one‐route nucleation method for perovskite nanocrystal synthesis was developed to improve the uniformity and stability of perovskite NCs. The activated PbBr_2_ precursors were controlled with high dissociation constant acids and bases. Uniform nanocrystal nucleation and growth were achieved. A novel resurface agents were synthesized to suppress Ostwald ripening and improve perovskite nanocrystal stability. Stable and uniform green LHP NCs were synthesized. The nanocrystal film exhibits excellent stability in the ambient environment (humidity 80%) and strong photostability under UV 365. The fabricated green PeQLEDs with ultra‐narrow emission (FWHM of 16.1 nm) achieved maximum EQE of 24.13% and lifetime T_50_ of 54 min at 10 000 cd m^−2^. The homogenous nucleation and growth synthesis reported can further lead to improving the quality and uniformity of LHP NCs for efficient and stable QLEDs.

## Conflict of Interest

The authors declare no conflict of interest.

## Author Contributions

X.Z. and Q.W. Contributed equally to this work. X. Z. and C. X. conceived the study. X. Z. developed synthesis method, Q.W. fabricated light‐emitting devices, and performed EQE and stability tests. X. Z. characterized perovskite properties. Z. Y. & M.D. perovskite synthesis, device fabrication and device optimization. Y.R. and J. W. assisted perovskite characterization and data analysis. C. X. and L. Q. supervised the project. Y.Y. helped with data analysis and revised the manuscript. All authors discussed the results and commented on the paper.

## Supporting information

Supporting Information

## Data Availability

The data that support the findings of this study are available from the corresponding author upon reasonable request.

## References

[1] T.‐H. Han , K. Y. Jang , Y. Dong , R. H. Friend , E. H. Sargent , T.‐W. Lee , Nat. Rev. Mater. 2022, 7, 757.

[2] A. Dey , J. Ye , A. De , E. Debroye , S. K. Ha , E. Bladt , A. S. Kshirsagar , Z. Wang , J. Yin , Y. Wang , L. N. Quan , F. Yan , M. Gao , X. Li , J. Shamsi , T. Debnath , M. Cao , M. A. Scheel , S. Kumar , J. A. Steele , M. Gerhard , L. Chouhan , K. Xu , X.‐G. Wu , Y. Li , Y. Zhang , A. Dutta , C. Han , I. Vincon , A. L. Rogach , et al., ACS Nano 2021, 15, 10775. 34137264 10.1021/acsnano.0c08903 PMC8482768

[3] M. I. Bodnarchuk , S. C. Boehme , S. ten Brinck , C. Bernasconi , Y. Shynkarenko , F. Krieg , R. Widmer , B. Aeschlimann , D. Günther , M. V. Kovalenko , I. Infante , ACS Energy Lett. 2019, 4, 63. 30662955 10.1021/acsenergylett.8b01669 PMC6333230

[4] C. Bi , Z. Yao , X. Sun , X. Wei , J. Wang , J. Tian , Adv. Mater. 2021, 33, 2006722. 10.1002/adma.202006722 33629762

[5] J.‐N. Yang , T. Chen , J. Ge , J.‐J. Wang , Y.‐C. Yin , Y.‐F. Lan , X.‐C. Ru , Z.‐Y. Ma , Q. Zhang , H.‐B. Yao , J. Am. Chem. Soc. 2021, 143, 19928. 34766754 10.1021/jacs.1c09948

[6] J. Cao , C. Yan , C. Luo , W. Li , X. Zeng , Z. Xu , X. Fu , Q. Wang , X. Chu , H. Huang , X. Zhao , J. Lu , W. Yang , Adv. Opt. Mater. 2021, 9, 2100300.

[7] L. Protesescu , S. Yakunin , M. I. Bodnarchuk , F. Krieg , R. Caputo , C. H. Hendon , R. X. Yang , A. Walsh , M. V. Kovalenko , Nano Lett. 2015, 15, 3692. 25633588 10.1021/nl5048779 PMC4462997

[8] Y. Dong , T. Qiao , D. Kim , D. Parobek , D. Rossi , D. H. Son , Nano Lett. 2018, 18, 3716. 29727576 10.1021/acs.nanolett.8b00861

[9] J. Song , J. Li , X. Li , L. Xu , Y. Dong , H. Zeng , Adv. Mater. 2015, 27, 7162. 26444873 10.1002/adma.201502567

[10] T. Fang , T. Wang , X. Li , Y. Dong , S. Bai , J. Song , Sci. Bull. 2021, 66, 36. 10.1016/j.scib.2020.08.025 36654311

[11] Y. Dong , Y.‐K. Wang , F. Yuan , A. Johnston , Y. Liu , D. Ma , M.‐J. Choi , B. Chen , M. Chekini , S.‐W. Baek , L. K. Sagar , J. Fan , Y. Hou , M. Wu , S. Lee , B. Sun , S. Hoogland , R. Quintero‐Bermudez , H. Ebe , P. Todorovic , F. Dinic , P. Li , H. T. Kung , M. I. Saidaminov , E. Kumacheva , E. Spiecker , L.‐S. Liao , O. Voznyy , Z.‐H. Lu , E. H. Sargent , Nat. Nanotechnol. 2020, 15, 668. 32632321 10.1038/s41565-020-0714-5

[12] C. Qin , A. S. D. Sandanayaka , C. Zhao , T. Matsushima , D. Zhang , T. Fujihara , C. Adachi , Nature 2020, 585, 53. 32879501 10.1038/s41586-020-2621-1

[13] Q. Chen , J. Wu , X. Ou , B. Huang , J. Almutlaq , A. A. Zhumekenov , X. Guan , S. Han , L. Liang , Z. Yi , J. Li , X. Xie , Y. Wang , Y. Li , D. Fan , D. B. L. Teh , A. H. All , O. F. Mohammed , O. M. Bakr , T. Wu , M. Bettinelli , H. Yang , W. Huang , X. Liu , Nature 2018, 561, 88. 30150772 10.1038/s41586-018-0451-1

[14] Q. A. Akkerman , G. Rainò , M. V. Kovalenko , L. Manna , Nat. Mater. 2018, 17, 394. 29459748 10.1038/s41563-018-0018-4

[15] Q. A. Akkerman , T. P. T. Nguyen , S. C. Boehme , F. Montanarella , D. N. Dirin , P. Wechsler , F. Beiglböck , G. Rainò , R. Erni , C. Katan , J. Even , M. V. Kovalenko , Science 2022, 377, 1406. 36074820 10.1126/science.abq3616

[16] G. Almeida , L. Goldoni , Q. Akkerman , Z. Dang , A. H. Khan , S. Marras , I. Moreels , L. Manna , ACS Nano 2018, 12, 1704. 29381326 10.1021/acsnano.7b08357 PMC5830690

[17] R. Grisorio , E. Fanizza , I. Allegretta , D. Altamura , M. Striccoli , R. Terzano , C. Giannini , V. Vergaro , G. Ciccarella , N. Margiotta , G. P. Suranna , Nanoscale 2020, 12, 623. 31829364 10.1039/c9nr08079a

[18] S. Tu , M. Chen , L. Wu , J. Mater. Chem. C 2021, 9, 3715.

[19] X. Li , K. Zhang , J. Li , J. Chen , Y. Wu , K. Liu , J. Song , H. Zeng , Adv. Mater. Interfaces 2018, 5, 1800010.

[20] M. Koolyk , D. Amgar , S. Aharon , L. Etgar , Nanoscale 2016, 8, 6403. 26841055 10.1039/c5nr09127f

[21] J. De Roo , M. Ibáñez , P. Geiregat , G. Nedelcu , W. Walravens , J. Maes , J. C. Martins , I. Van Driessche , M. V. Kovalenko , Z. Hens , ACS Nano 2016, 10, 2071 26786064 10.1021/acsnano.5b06295

[22] Y.‐H. Kim , S. Kim , A. Kakekhani , J. Park , J. Park , Y.‐H. Lee , H. Xu , S. Nagane , R. B. Wexler , D.‐H. Kim , S. H. Jo , L. Martínez‐Sarti , P. Tan , A. Sadhanala , G.‐S. Park , Y.‐W. Kim , B. Hu , H. J. Bolink , S. Yoo , R. H. Friend , A. M. Rappe , T.‐W. Lee , Nat. Photonics 2021, 15, 148.

[23] M. Imran , P. Ijaz , D. Baranov , L. Goldoni , U. Petralanda , Q. Akkerman , A. L. Abdelhady , M. Prato , P. Bianchini , I. Infante , L. Manna , Nano Lett. 2018, 18, 7822 30383965 10.1021/acs.nanolett.8b03598 PMC6428374

[24] B. M. Cossairt , Chem. Mater. 2016, 28, 7181.

[25] S. Huang , Z. Li , B. Wang , N. Zhu , C. Zhang , L. Kong , Q. Zhang , A. Shan , L. Li , ACS Appl. Mater. Interfaces 2017, 9, 7249. 28181794 10.1021/acsami.6b14423

[26] X. Peng , Nano Res. 2009, 2, 425.

[27] A. Dutta , S. K. Dutta , S. Das Adhikari , N. Pradhan , ACS Energy Lett. 2018, 3, 329.

[28] F. Krieg , S. T. Ochsenbein , S. Yakunin , S. Ten Brinck , P. Aellen , A. Süess , B. Clerc , D. Guggisberg , O. Nazarenko , Y. Shynkarenko , S. Kumar , C.‐J. Shih , I. Infante , M. V. Kovalenko , ACS Energy Lett. 2018, 3, 641. 29552638 10.1021/acsenergylett.8b00035 PMC5848145

[29] D. Quarta , M. Imran , A.‐L. Capodilupo , U. Petralanda , B. Van Beek , F. De Angelis , L. Manna , I. Infante , L. De Trizio , C. Giansante , J. Phys. Chem. Lett. 2019, 10, 3715 31244273 10.1021/acs.jpclett.9b01634

[30] F. Krieg , P. C. Sercel , M. Burian , H. Andrusiv , M. I. Bodnarchuk , T. Stöferle , R. F. Mahrt , D. Naumenko , H. Amenitsch , G. Rainò , M. V. Kovalenko , ACS Cent. Sci. 2021, 7, 135 33532576 10.1021/acscentsci.0c01153 PMC7845019

[31] D. Yang , X. Li , W. Zhou , S. Zhang , C. Meng , Y. Wu , Y. Wang , H. Zeng , Adv. Mater. 2019, 31, 1900767. 10.1002/adma.201900767 31172615

