# Supplementary material for: Stable Perovskite Quantum Dots Light‐Emitting Diodes with Efficiency Exceeding 24%
Source: Adv Sci (Weinh). 2023 Oct 27;10(36):2304696. doi: 10.1002/advs.202304696 (PMC10754115; doi:10.1002/advs.202304696)
Supplement: Supplementary file 1 — Supporting Information [file ADVS-10-2304696-s001.pdf]

ADVANCED SCIENCE
Open Access

Supporting Information

for *Adv. Sci.*, DOI 10.1002/advs.202304696

Stable Perovskite Quantum Dots Light-Emitting Diodes with Efficiency Exceeding 24%

Xuanyu Zhang, Qiangqiang Wang, Zhiwei Yao, Ming Deng, Jing Wang, Lei Qian, Yong Ren, Yuying Yan* and Chaoyu Xiang**

Supporting Information: Stable Perovskite Quantum Dots Light-emitting Diodes with Efficiency Exceeding 24%

Xuanyu Zhang[1,2,4,5,6], Qiangqiang Wang[1,4,5,9] Zhiwei Yao[1,4,5], Ming Deng[1,4,5], Jing Wang[7,8], Lei Qian[1,4,5,6], Yong Ren[2,7]*, Yuying Yan[3]*, Chaoyu Xiang[1,4,5,6]*

1. Laboratory of Advanced Nano-Optoelectronic Materials and Devices, Ningbo Institute of Materials Technology and Engineering, Chinese Academy of Science, Ningbo, Zhejiang, 315201, China
2. Nottingham Ningbo China Beacons of Excellence Research and Innovation Institute, Ningbo 315040, China
3. Faculty of Engineering University of Nottingham, Nottingham, UK
4. Division of Functional Materials and Nanodevices, Ningbo Institute of Materials Technology and Engineering, Chinese Academy of Sciences, Ningbo 315201, China
5. Laboratory of Advanced Nano-Optoelectronic Materials and Devices, Qianwan Institute of CNITECH, Ningbo, P. R. China, Ningbo 315300, China.
6. Zhejiang Provincial Engineering Research Center of Energy Optoelectronic Materials and Devices, Ningbo Institute of Materials Technology & Engineering, Chinese Academy of Sciences.
7. Key Laboratory of Carbonaceous Wastes Processing and Process Intensification Research of Zhejiang Province, University of Nottingham Ningbo China, Ningbo 315100, China
8. Department of Electrical and Electronic Engineering, University of Nottingham Ningbo China, Ningbo 315100, China
9. School of Mechanical Engineering and Mechanics, Ningbo University, Ningbo, Zhejiang 315211, China

Materials. Cesium carbonate (Cs_2CO_3, Sigma-Aldrich 99.995%), oleylamine (OAm, Aladdin 90%), octanoic acid (OTAc, Aladdin, 99%), octylamine (OTAm, Aladdin, 99%), octylphosphonic acid (OPA, Aladdin, 97%) lead (II) bromide ($PbBr_2$ Aladdin 99.999%), 4-Fluorophenethylamine (4-FPEA, Aladdin 90%), 1-octadecene (ODE, Sigma-Aldrich 90%), didodecylamine (DDDAM, TCI 97%), hydrogen bromide (HBr, alfa), hexane (Aladdin 99%), methyl acetate (MeOAc, Aladdin anhydrous 99.5%), oleic acid (OA, Sigma-Aldrich 90%), ethyl acetate (Aladdin anhydrous 99.5%), 3-(N,N-dimethyloctadecylammonio) propanesulfonate (ASC18, Aladdin, 99.8%), octane (Aladdin 99%), mesitylene (Aladdin 99%), chlorobenzene (CB, Mackin, 99.8%), polymethylmethacrylate (PMMA, Sigma-Aldrich), (1,3,5-Benzinetriyl)-tris(1-phenyl-1H-benzimidazole) (TPBi), 2,4,6-tris(3-(diphenylphosphoryl)phenyl)-1,3,5-triazine (PO-T2T, 99.5%), Lithium fluoride (LiF) are from Xi'an Polymer Light Technology.

Preparations of Cs-oleate precursor. 1.5 mmol of Cs_2CO_3, and 9 mL ODE with 1 mL OA were placed together in a 50 ml three-necked round bottom flask. The mixture solution was heated to 40 ℃ and vacuum dried for 30min. Then, the temperature was increased to 100 ℃, and continued vacuum drying for 1 hour. Then argon was pumped into the flask and continued degassing for 30 min. After completely removing H_2O and O_2. The clear Cs-Oleate solution was obtained.

Synthesis of OA/OAm $CsPbBr_3$ nanocrystals. In a 50 ml three-necked flask, 138 mg $PbBr_2$ with 10 ml ODE was vacuum dried at 40 ℃ for 30 minutes. Then, the temperature was increased to 100 ℃, and continued vacuum drying for 1 hour. The argon flow was pumped into the flask and

kept the lead halide precursor in the argon atmosphere. 1 ml OA and 1 ml OAm were injected into the mixtures to dissolve the $PbBr_2$. After the $PbBr_2$ was completely dissolved and formed a clear solution, the temperature was increased to 160 ℃. 0.8 ml Cs-oleate precursor solution was quickly injected into the flask at 160 ℃. After 5 seconds, the flask was swiftly cooled by an ice water bath. The nanocrystals were first purified using methyl acetate. Ethyl acetate was used for the second purification.

Synthesis of ASC18-HBr. In a 50 ml three-necked flask, 172 mg ASC18 with 4 ml mesitylene was degassed using argon flow for 30 min at room temperature. 100 μL of HBr was injected and the temperature was elevated to 130 ℃ in the argon atmosphere. The reaction between ASC18 and HBr was continued for 1 hour and formed a transparent rufous solution. The temperature of the ASC-HBr solution was kept at 130 ℃ before use.

The OTAc/OTAm $CsPbBr_3$ nanocrystals synthesis. The procedures for drying materials were the same. The OA and OAm for dissolving $PbBr_2$ in typical synthesis were replaced by a mixture of OA,OAm, OTAc, and OTAm here (for OA/OAm passivated nanocrystals, no OTAc and OTAm were used). 0.3 ml OA, 0.3 ml OAm, 0.4 ml OTAc, and 0.4 ml OTAm were injected into the mixture for dissolving $PbBr_2$ at 100 ℃. After 0.8 ml Cs-oleate precursor solution was quickly injected into the flask at 160 ℃. 2 ml of ASC18-HBr mixture was injected into the flask, followed by injection of 4-FPEA in toluene. After 5 seconds, the flask was swiftly cooled by an ice water bath. The nanocrystals were first purified using methyl acetate as an anti-solvent (volume ratio 1:2). The nanocrystals were dispersed with 4ml toluene. 80 μL OPA toluene solution

(0.25mmol/ml) was added for ligand exchange. Ethyl acetate was used for the second purification (volume ratio 1:2.5) and nanocrystals were redispersed with octane.

Device Fabrications: The ITO pattern glass substrate was cleaned with acetone, isopropanol, and ethanol in turn. Each time, it was ultrasonic for 15 minutes. After cleaning, it was treated with ultraviolet ozone for 15 minutes to adjust the ITO work function. An aqueous solution of PEDOT: PSS was spin-coated on ITO glass substrate at 2000 rpm for 40 s, and then annealed at 150 ℃ for 30 minutes; Then, it was transferred to the glove box with nitrogen, 8 mg/mL PTAA chlorobenzene solution was spin-coated onto the PEDOT: PSS layer and bake for 15 min at 120 °C. PMMA (3 mg/mL) was spin-coated at 5000 rpm for 30 s and then annealed at 120 ℃ for 10 min. 25 mg/mL perovskite nanocrystal octane solution was spin-coated at 2000 rpm for 30 s, and then annealed at 60 ℃ for 10 min. 5 nm TPBi,35 nm PO-T2T, 1 nm LiF and 100 nm Al electrodes were evaporated under $2*10^{-4}$ Pa pressure.

UV-Vis UV-vis absorption spectra were obtained by Perkin-Elmer Lambda 950.

TEM TEM and HRTEM of nanocrystals were obtained by a Talos F200X with 200 kV acceleration voltage.

In-situ PL A monochromatic source (LED) was used as an excitation source for all PL measurements. The emission originating from the sample coupled via a 10x objective to a fiber spectrometer through a 2 m long multimode fiber with a core diameter of 400 µm. The spectrometer comprised a 20 µm entrance slit, a 600 lines/mm grating, and a detector containing 2048 pixels. The spectrometer was operated between 400 and 1100 nm, and data was recorded

using 10–100 ms integration times. The entire system was enclosed in a black box to minimize pollution from the stray light.

PL Photoluminescence spectra profiles were obtained by Horiba FL3-111 with an excitation source at 400 nm and 320 nm.

PLQY Photoluminescence quantum yield was obtained by Otsuka QE2100.

TRPL Time-resolved PL decay spectra of nanocrystals were obtained by Horiba FL3-111.

XRD The XRD spectra of nanocrystal film were obtained by a Bruker D8 Advanced Davinci using Cu Kα radiation (λ = 1.5418 Å).

FTIR FTIR spectra were collected using a Cary660+620 attenuated total reflectance FTIR system. All nanocrystal solid film samples were cast on glass substrates.

XPS measurements. XPS measurements were performed using an ESCALAB 250Xi with a base pressure of ~10^{-9} torr. The X-ray radiation is Al Kα emission (1486.7 eV, take-off angle, 75°).

Device characterization. The electroluminescence performance of devices was measured with the same setup reported before[1]. Electroluminescence spectra were obtained using an Ocean Optics USB 2000+ spectrometer with the devices driven at a constant current with a Keithley 2400 source meter. The J-L-V characteristics of the devices were taken under ambient conditions with a Keithley 2400 source meter measuring the sweeping voltages and currents and a Keithley 6485 Picoammeter together with a calibrated silicon detector (Edmund) measuring light intensities. Luminance was calibrated using a photometer (Spectra Scan PR655) with the assumption of Lambertian emission pattern of all devices. The operational lifetime test was conducted under ambient conditions at room temperature (22±2°C) using a commercialized lifetime test system (Guangzhou Jinghe Equipment Co., Ltd). The devices were encapsulated with Nagase UV epoxy resin XNR5516Z(C)-SA1 and capping glasses.

Lifetime Test Lifetime of PeLEDs was measured through a commercialized lifetime test system (Guangzhou Jinghe Equipment Co., Ltd) under ambient conditions.

Photostability test Intensity of UV 365 used for this test is 320 mW/cm^2 and 500 mW/cm^2.

Defects density and hole mobility calculation

The defect density of thin films could be calculated by trap-filled limit region (V_{TFL}) as shown in equation (1). The hole mobility of perovskite nanocrystal films can be calculated by the Mott-Gurney law equation (2).

$$N_t = \frac{2\varepsilon\varepsilon_0 V_{TFL}}{ed^2} \quad (1)$$

$$J_D = \frac{9\varepsilon\varepsilon_0 \mu V^2}{8d^3} \quad (2)$$

The structure of our hole-only devices is ITO/PEDOT: PSS (30 nm)/PTAA (35 nm)/PMMA/QDs (~240 nm) /MoO_3 (10 nm)/Ag. N_t is the trap state density, V_{TFL} is the trap-filled limit voltage, d is the distance between the electrodes, e is the elementary charge, ε_0 and ε are the vacuum permittivity and relative permittivity, respectively. μ is carrier mobility which is hole mobility here. J_D and V are current density and bias in Child's law region.

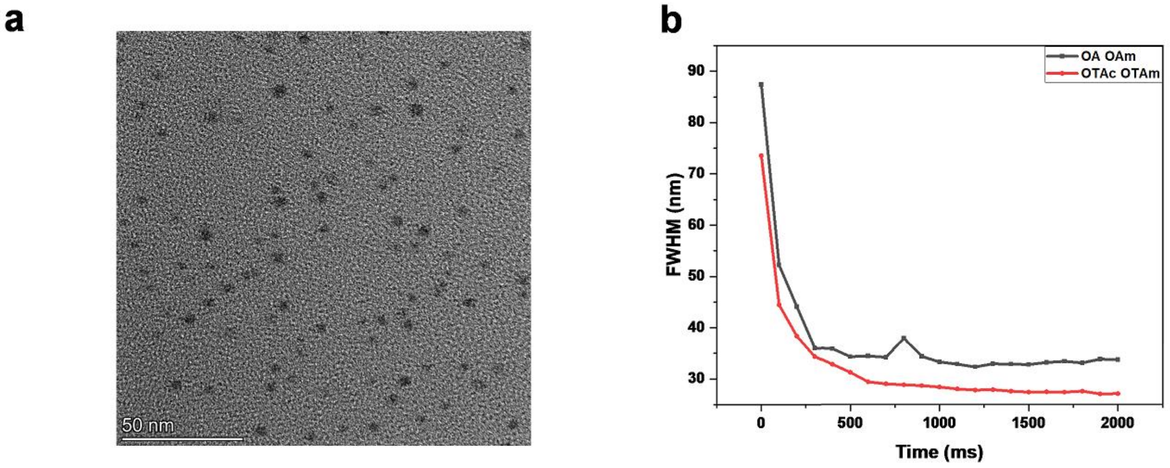

Figure S1 (a) TEM image of $PbBr_2$ clusters (b) PL FWHM evolution in the synthesis process of OA/OAm, OTAc/OTAm

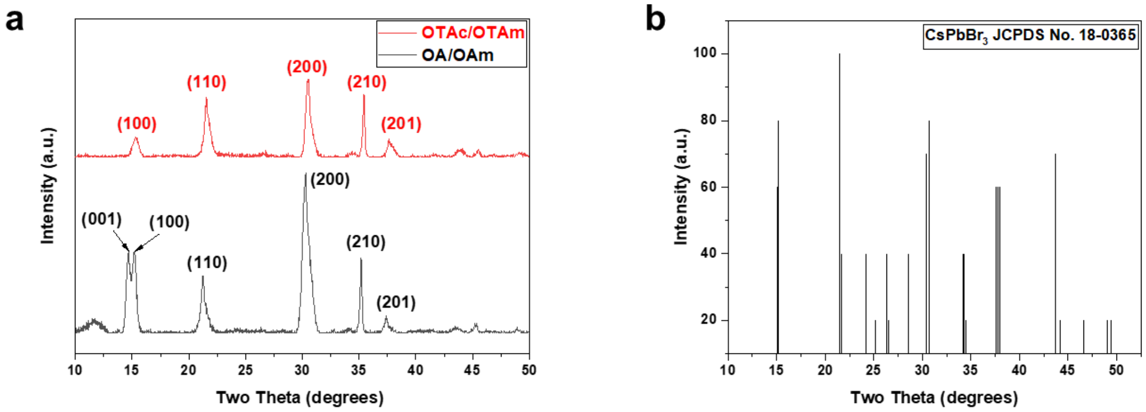

Figure S2 XRD pattern of OTAc/OTAm and OA/OAm nanocrystals film on ITO glass

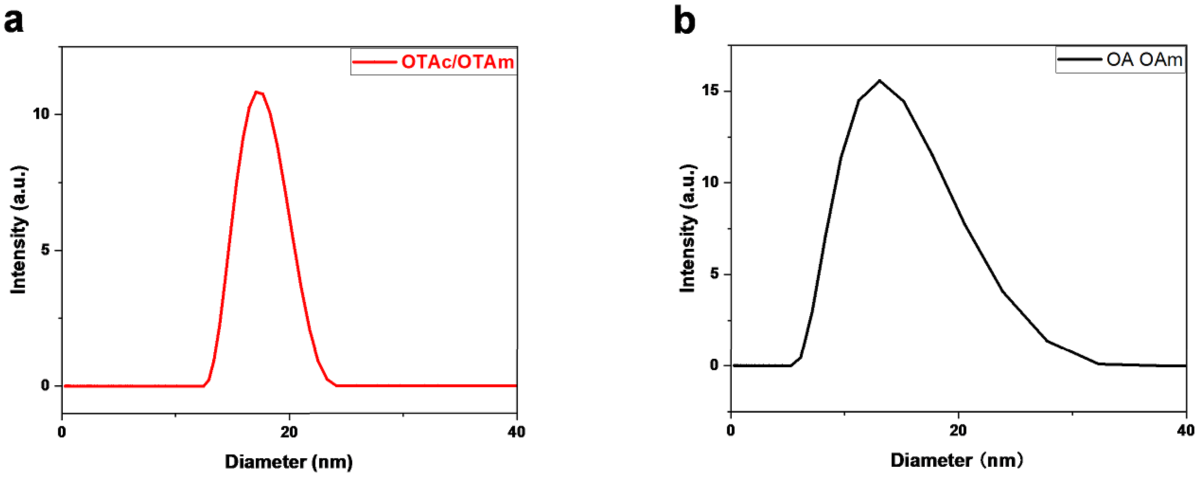

Figure S3 Measured DLS size distribution of perovskite nanocrystals, (a) OTAc/OTAm ,(b) OA/OAm.

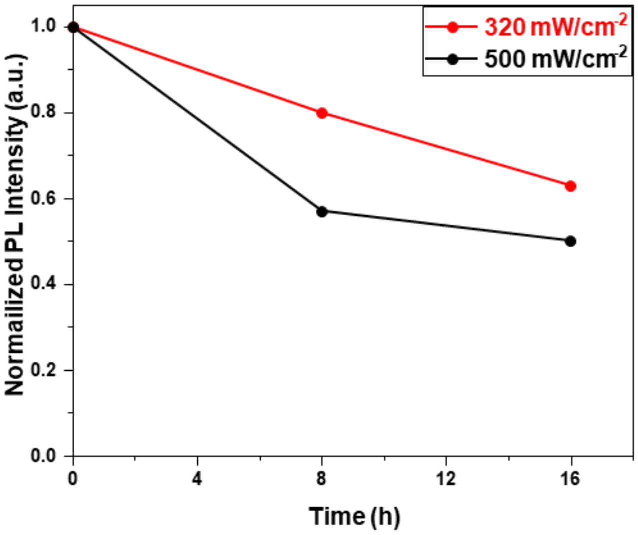

Figure S4 PL intensity decay of OTAc/OTAm perovskite films under different intensity of UV 365

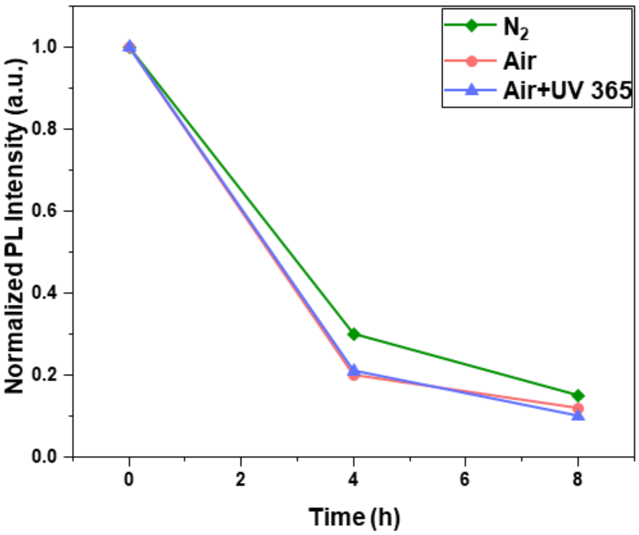

Figure S5 PL intensity decay of OA/OAm perovskite films under different conditions

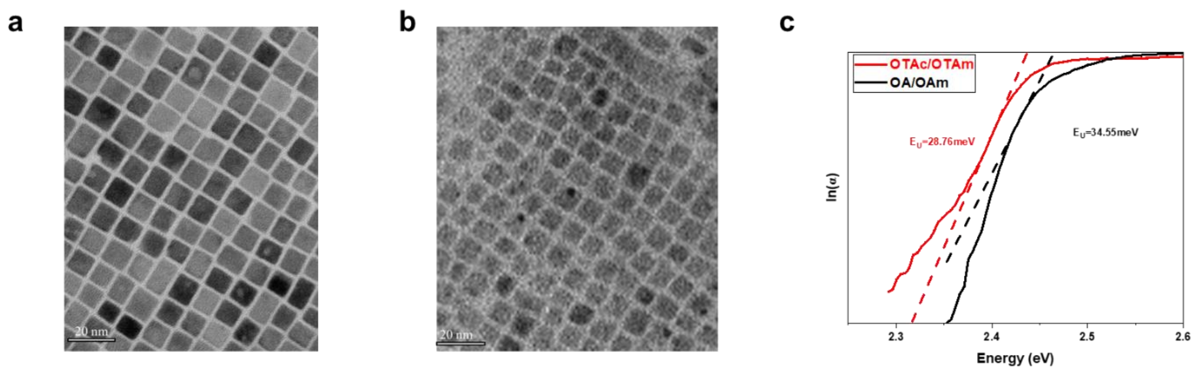

Figure S6 TEM image of purified perovskite nanocrystals, (a) OTAc/OTAm, (b) OA/OAm. (c) Urbach absorption edge of OTAc/OTAm and OA/OAm perovskite nanocrystals.

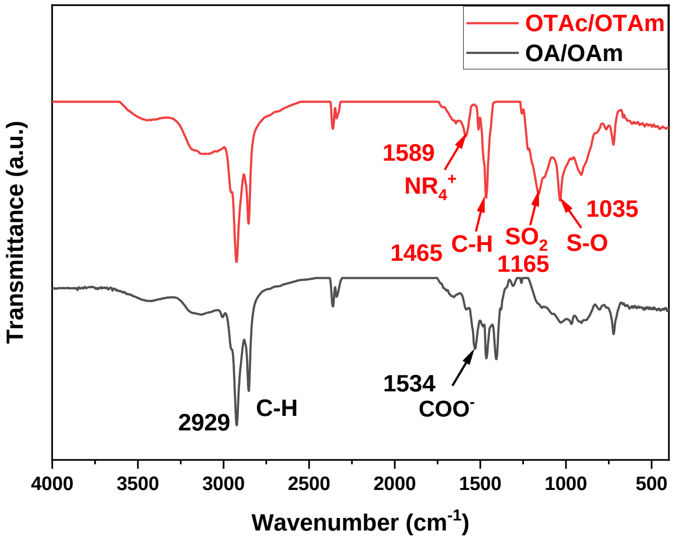

Figure S7 Comparison of FTIR characterization of perovskite nanocrystal films

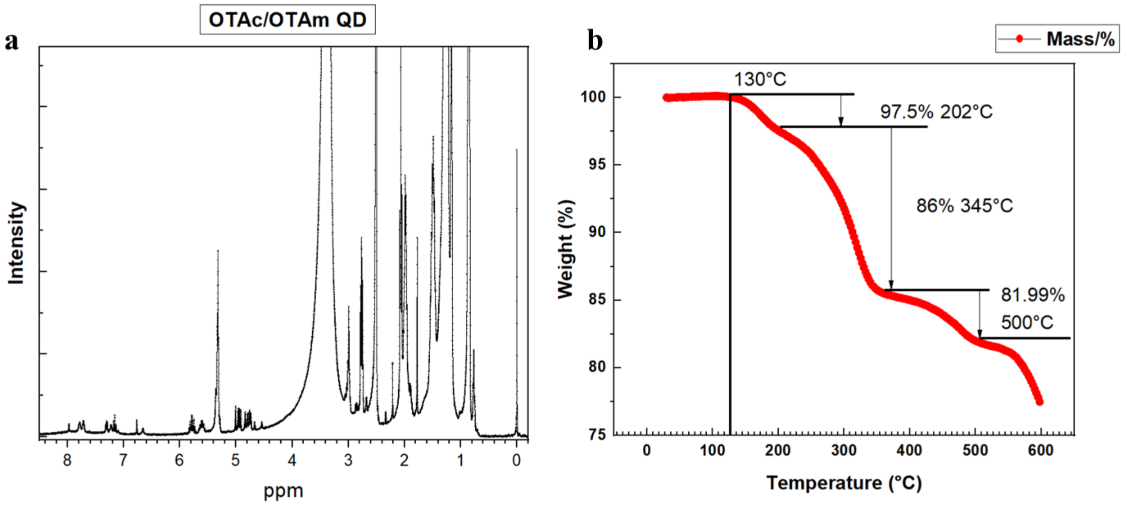

Figure S8 (a) ^{1}H-NMR characterization of OTAc/OTAm perovskite nanocrystals (b)TGA measurement of synthesized OTAc/OTAm perovskite nanocrystals

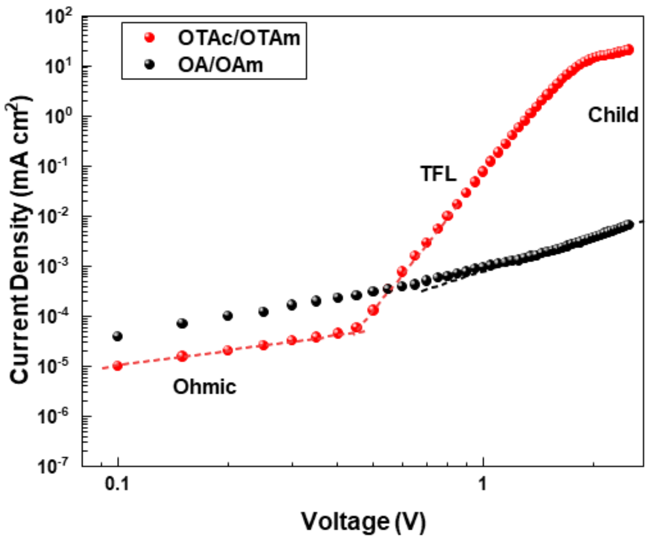

Figure S9 J-V characteristic of hole-only devices for OTAc/OTAm and OA/OAm perovskite nanocrystals

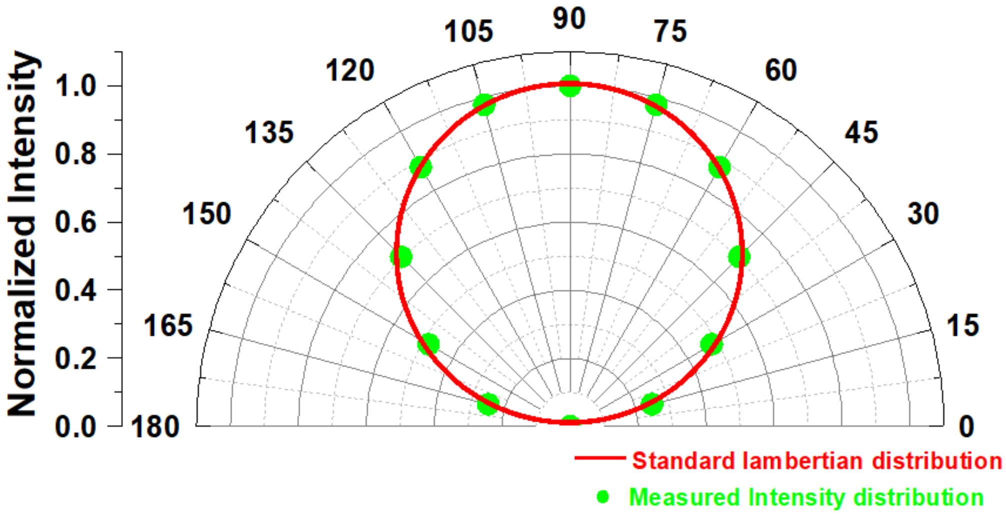

Figure S10 Angular intensity distribution of fabricated LEDs

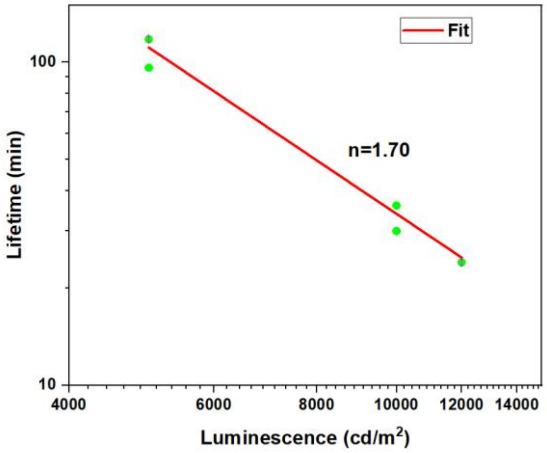

Figure S11 Initial luminescence value versus T_{50} measured of PeQLEDs

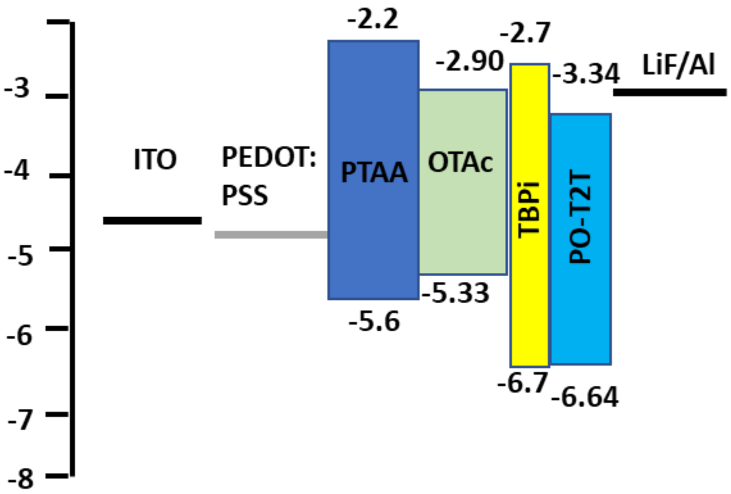

Figure S12 Energy diagram of fabricated PeQLEDs

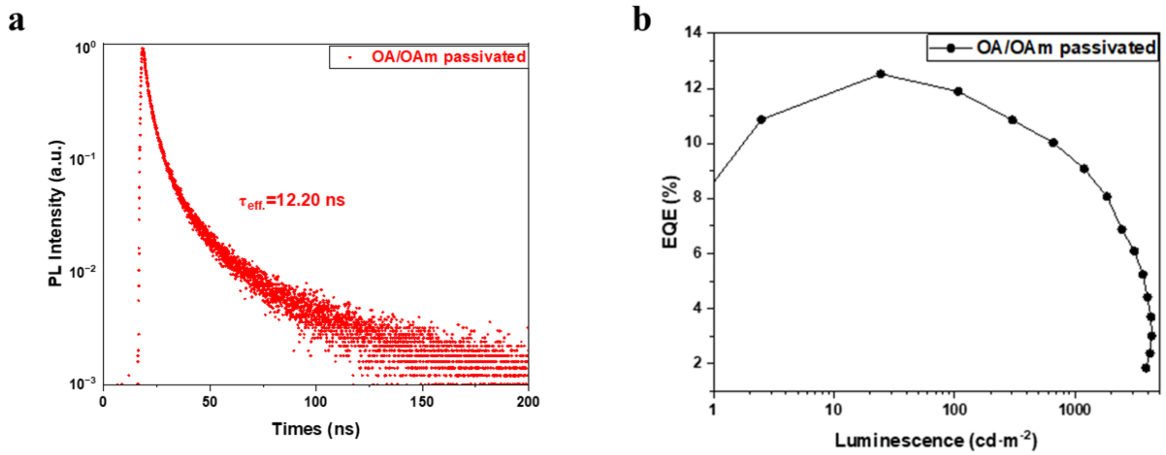

Figure S13 OA/OAm nanocrystals passivated with strong ligands (ASC18-HBr, 4-FPEA, OPA): (a) TRPL of perovskite nanocrystal film, (b) EQE versus luminescence curve of PeQLED.

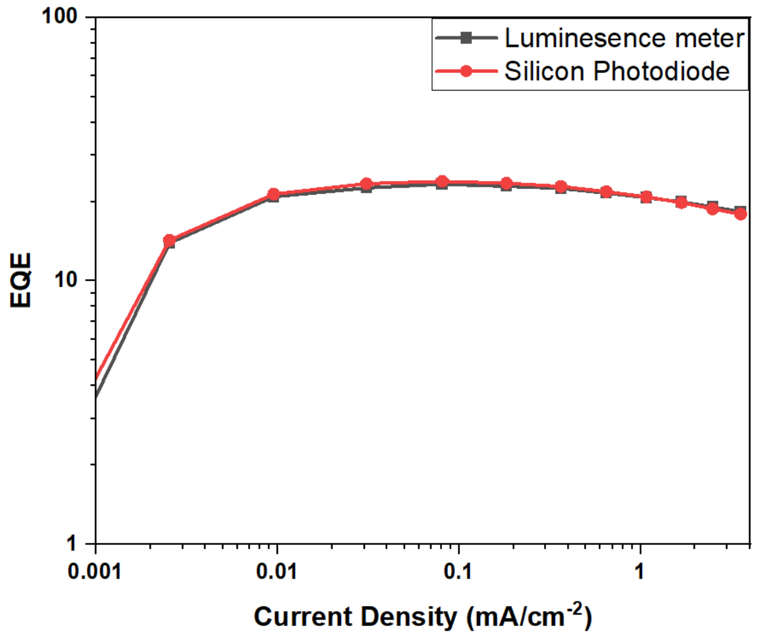

Figure S14 Measured EQE of OTAm/OTAc PeQLED with different setups.

Table S1 Parameters used for fitting the PL decay of perovskite nanocrystal film.

	τ1(ns)	τ2(ns)	τ3(ns)	B_1	B_2	B_3	$\tau_{eff.}$(ns)
OTAc/OTAm LHP film	14.22	73.14	2.75	1844.703	190.3808	5666.055	25.75
OA/OAm LHP film passivated	6.88	31.34	1.90	1552.925	199.0967	3194.595	12.20
OA/OAm LHP film	3.75	23.32		7847.748	162.2948		5.98

Table S2 Comparison of EQE and a lifetime of high-performance PeQLEDs

QDs Structure	Maximum EQE	Maximum Luminescence	Operation Lifetime	Reference
$CsPbBr_3$	22.00%	10000cd/m^2	T_{50}_1200 nits 60 mins	Nat. Nanotechnology. 15, 668–674 (2020).
$CsPbBr_3$	19.30%	2000cd/m^2	T_{50}_1000 nits 50s	Nat. Photonics 15, 379–385 (2021)
$CsPbBr_3$	21.63%	41900cd/m^2	T_{50}_4000 nits 69 mins	Science Bulletin 66 (2021) 36-43
$FAGAPbBr_3$	23.40%	25000cd/m^2	T_{50}_100 nits 132 mins	Nat. Photonics 15, 148–155 (2021)
$CsPbBr_3$	21.60%	36000cd/m^2	T_{50}_1000 nits 25.3 mins	Nature 612, 679–684 (2022).
$CsPbBr_3$	17.85%	10000 cd/m^2	T_{50}_100 nits 177 mins	Journal of the American Chemical Society 2021 143 (47), 19928-19937
$FAPbBr_3$	19.20%	67115cd/m^2	T_{50}_100 nits 20 mins	ACS Energy Lett. 2021, 6, 2395−2403
**$CsPbBr_3$**	**24.13%**	**33009cd/m^2**	**T_{50}_1000 nits 2706 mins**	**This work**

Table S3 Comparison of PL stability of perovskite nanocrystal film

Material	PL Stability	Reference
CsPbBr3 QDs film	50% of initial QY after 400 mins (UV365+RH.70%)	Angew. Chem. Int. Ed. 2023, e202303462
CsPbBr3 QDs film	30% of initial QY after 960 mins (UV254+RH.30%)	Nat. Photonics 15, 843-849 (2021)
CsPbBr3 film	~35% of initial QY after 600 mins (RH.50%)	Adv. Mater 33, 2103268 (2021)
CsPbBr3 QDs film	90% of initial QY after 960 mins (UV365+RH.80%)	This work

Reference

1. Xiang, C. et al. High efficiency and stability of ink-jet printed quantum dot light emitting diodes. Nat. Commun. 11, 1646 (2020).
